# Mr. Brown, on the Contagious Nature of Yellow Fever

**Published:** 1806-06

**Authors:** David Brown

**Affiliations:** Surgeon Royal Artillery, on the Jamaica Station. Port Royal, Jamaica


### *To the Editors of the Medical and Physical Journal.*

GENTLEMEN,

I Find that the Paper of Doctor Dancer's, on the contagious nature of malignant Yellow Fever, has given great umbrage to Mr. Alder:—who this gentleman is, I declare I do not know, nor have I ever seen his celebrated Out-lines; but from the out-line he has given of himself, in this unprovoked attack upon me, I am apt to imagine his mind is discomposed by a contagion of some kind or other. I leave it to you, Mr. Editors, or to any impartial person, to say, whether there be any abuse or personality in the paper referred to; if there had, for the credit of your Journal, you would not have inserted it; and the remarks Mr. Alder has chosen to construe in that sense, are not mine, but Doctor Dancer's, who, I verily believe, is as innocent as myself, of any acquaintance with the writings of Mr. Alder.

The opinion of Mr. Alder, that contagion can be conveyed by the sea-breeze, to the distance of fifty miles; nay, that it can be borne on the wings of the wind, across the ocean, from St. Domingo to Up Park Camp in Jamaica, is so singular, that I shall forbear any comment on it, wishing to decline a combat, where victory would be no triumph. I am, &c.

DAVID BROWN.

Surgeon Royal Artillery, on the Jamaica Station.

*Port Royal, Jamaica,*
*March* 29, 1806.

